# Psycho-Therapeutics; or, Treatment by Hypnotism and Suggestion

**Published:** 1892-06

**Authors:** 


*Psycho-Therapeutics; or, Treatment by Hypnotism and Suggestion.* By C. LLOYD TUCKEY, M.D. Third Edition. Pp. xvi., 321. London: BAILLIÈRE, TINDALL AND COX. 1891.

*The Practice of Hypnotic Suggestion.* By GEO. KINGSBURY, M.A., M.D. Pp. viii., 206. Bristol: JOHN WRIGHT & CO. 1891.

These two volumes are, with the exception of one by Dr. Felkin recently noticed in this *Journal*, the only books as yet written by English authors on the subject of hypnotism. The former work, which has been greatly added to since its first appearance, is a bulky, fairly clear review of the many observations on, and theories concerning, the nature of the hypnotic state. The latter is, as its title implies, a short practical guide, which may be recommended to those who wish to test for themselves the therapeutic uses of suggestion during hypnosis in certain functional nervous disorders. Both books are of interest and value, in that they give an account of cases treated by the authors; for we have been so dependent on our Continental *confrères* for our knowledge of the subject that most Englishmen who have not seen the hypnotic state class it with theosophic phenomena and Stead's ghost stories.

